# Novel Microcrystal Formulations of Sorafenib Facilitate a Long-Acting Antitumor Effect and Relieve Treatment Side Effects as Observed With Fundus Microcirculation Imaging

**DOI:** 10.3389/fonc.2021.743055

**Published:** 2021-08-26

**Authors:** Junxiao Wang, Rui Liu, Yun Zhao, Zhenhu Ma, Zejie Sang, Zhenyu Wen, Xueling Yang, Hui Xie

**Affiliations:** ^1^Department of Interventional Radiology, Senior Department of Oncology, The Fifth Medical Center of People’s Liberation Army (PLA) General Hospital, Beijing, China; ^2^Department of Occupational and Environmental Health, School of Public Health, Jilin University, Changchun, China; ^3^Department of Medical Oncology, Senior Department of Oncology, the Fifth Medical Center of PLA General Hospital, Beijing, China; ^4^Department of Interventional Therapy, Tianjin Medical University Cancer Institute & Hospital/National Clinical Research Center for Cancer, Tianjin’s Clinical Research Center for Cancer/Key Laboratory of Cancer Prevention and Therapy, Tianjin, China

**Keywords:** advanced hepatocellular carcinoma, novel sorafenib microcrystal, sustained release and long acting, fundus microcirculation imaging, molecular targeted agents

## Abstract

The tyrosine kinase inhibitors (TKIs), including sorafenib, remain one first-line antitumor treatment strategy for advanced hepatocellular carcinoma (HCC). However, many problems exist with the current orally administered TKIs, creating a heavy medical burden and causing severe side effects. In this work, we prepared a novel microcrystalline formulation of sorafenib that not only achieved sustainable release and long action in HCC tumors but also relieved side effects, as demonstrated by fundus microcirculation imaging. The larger the size of the microcrystalline formulation of sorafenib particle, the slower the release rates of sorafenib from the tumor tissues. The microcrystalline formulation of sorafenib with the largest particle size was named as Sor-MS. One intratumor injection (once administration) of Sor-MS, but not Sor-Sol (the solution formulation of sorafenib as a control), could slow the release of sorafenib in HCC tumor tissues and in turn inhibited the *in vivo* proliferation of HCC or the expression of EMT/pro-survival–related factors in a long-acting manner. Moreover, compared with oral administration, one intratumor injection of Sor-MS not only facilitated a long-acting antitumor effect but also relieved side effects of sorafenib, avoiding damage to the capillary network of the eye fundus, as evidenced by fundus microcirculation imaging. Therefore, preparing sorafenib as a novel microcrystal formulation could facilitate a long-acting antitumor effect and relieve drug-related side effects.

## Introduction

Currently, hepatocellular carcinoma (HCC) remains one of the most important threats to the public health system of China because of the highly infectious rates of hepatitis viruses [e.g., hepatitis B virus (HBV) or hepatitis C virus (HCV)], and many patients often present with advanced stages of HCC at the initial diagnosis (1–5). The use of tyrosine kinase inhibitors (TKIs) or the oral administration of molecularly targeted agents, represented by sorafenib (sorafenib tosylate tablets), could prolong the overall or progression-free survival of patients (6–8). However, some problems associated with TKIs include the following: (1) Gastrointestinal–digestive function injury in compromised patients often attenuates the absorption of TKIs (9, 10). (2) The current strategy of daily oral administration of TKI tablets could induce the systemic distribution of TKIs throughout the entire body, leading to insufficient local concentrations of TKIs in HCC lesions (11, 12). (3) The high daily dose (> 800 mg every day) of TKIs such as sorafenib could induce not only a heavy financial burden but also side effects (13). Therefore, research about more effective therapeutic strategies to enhance the antitumor effect of TKIs and reduce their side effects is warranted.

How to improve the effects of molecularly targeted drugs such as sorafenib and concurrently alleviate their side effects is of great significance. Long-term use of sorafenib can cause skin rashes, diarrhea, increased blood pressure, and skin swelling (11, 14). The inhibitory effect of sorafenib on VEGFR (vascular endothelial growth factor receptor) and other RTKs (receptor tyrosine protein kinases) (6–8, 11) is the foremost mechanism causing these side effects. Existing animal models for sorafenib toxicity studies have many shortcomings: experimental animals cannot accurately reflect the various pathological changes in the human body, and patients with advanced HCC often have different degrees of liver fibrosis and cirrhosis, which are difficult to replicate at the animal level (15, 16). At the same time, many difficulties exist in tissue microcirculation–related research: The resolution of contrast-enhanced ultrasound can reflect the bleeding supply to a certain extent, but detection of microcirculation changes is limited (17, 18), and pathological analyses, such as hematoxylin and eosin (H&E) staining, cannot reflect the state of tissue microcirculation throughout the body in animals (19, 20).

Among the TKIs (tyrosine-kinase inhibitors) that treat HCC, sorafenib has been used widely and for a long time; thus, sorafenib is the best understood treatment and a logical choice for research (14, 21, 22). Analysis of the chemical features of sorafenib show that it is insoluble in water; a current formulation strategy provides sorafenib as sorafenib mesylate tablets (23–25). A microcrystal formulation is a pharmaceutical formulation that exchanges drug powder to microcrystals with diameters of 30-50 μm (26–28). Previously, the microcrystals have been used to improve the absorption of insoluble drugs *via* oral administration because, unlike drug powders, they can contact and mix with digestive fluid much easier (29, 30). Since sorafenib is insoluble in water, it can be prepared as a microcrystal formulation. The microcrystalline preparation of sorafenib is directly injected into the tumor tissue, and it can stay in the tumor tissue for a long time. The larger the particle-size of the microcrystalline formulation, the easier it is to stay inside the tissue for a long time. In tumor tissues, through the erosion of sorafenib microcrystals by tumor tissue cells, sorafenib is gradually released and kills tumor cells. At the same time, because sorafenib microcrystals are directly injected into the tumor tissue, it can protect normal tissues from damage. In this work, we prepared the pure-powders of sorafenib as a novel microcrystal formulation. This approach could overcome the insoluble features of sorafenib powder and condense the drug concentration in the tumor without affecting the surrounding tissue. We also used retinal/fundal imagine in small animals to examine whether one-dose administration of microcrystal formulation of sorafenib could achieve a long-acting antitumor effect and improve the side effects associated with sorafenib.

## Materials and Methods

### Cell Culture and Preparation of Sorafenib Formulations

MHCC97-H cells (a highly aggressive HCC cell line) purchased from the Type Culture Collection of the Chinese Academy of Sciences were cultured using DMEM with 10% FBS at 37°C with 5% CO_2_. The pure-powder formulation of sorafenib (purity > 99% by high performance liquid chromatography) was a gift from Dr. Xi He in the Fifth Medical Center, General Hospital of Chinese People’s Liberation Army of China (PLA). To make the sorafenib solution (Sor-Sol) formulation, sorafenib was first dissolved with sodium dodecyl sulphate, DMSO (Dimethyl sulfoxide), PEG400, or Tween 80 (all purchased from Sigma Aldrich Corporation, St. Louis, MO, USA) and then was diluted with physiological saline accompanied by ultrasonic or churning conditions (ultimate concentrations of DMSO, PEG400, or Tween 80: 1%, 4%, or 4%, respectively) (31–33). To prepare a microcrystal formulation of sorafenib, the pure-powder formulation was dispersed by aqueous solution with 6.25% Tween 80 (27). Next, the systems were mixed using magnetic stirring, and the microcrystal formulation of sorafenib was prepared with a MiniZeta machine (NETZSCH Machinery and Instruments Corporation, Germany) equipped with the grinding media of yttrium-stabilized zirconium oxide beads (0.6 mm in diameter) forming a coarse suspension of sorafenib. Then, the coarse suspension was transferred into the milling bowl, and the individual particle diameter of sorafenib in microcrystal formulation was controlled by the agitator speed (500 rpm for large individual-particle diameter; 1500 rpm for medium diameter; 3000 rpm for small diameter) (27). The sorafenib concentration in the solution formulation was almost 2 mg/mL; conversely, the sorafenib concentration in the microcrystal formulation could reach 30 mg/mL, according to LC-MS/MS (liquid chromatography tandem mass spectrometry/mass spectrometry) (34). To perform a comparison experiment between Sor-Sol and Sor-MS, Sor-MS needed to be diluted to achieve a matching sorafenib content of 2 mg/mL. The sorafenib microcrystal formulations were observed by an optical microscope and a conventional transmission electron microscope according to the methods of Yuan et al. in 2021 (35) and Quan et al. in 2020 (36). The particle size distribution charts were obtained as described in our previous publication (27).

Next, the microcrystalline formulation of sorafenib were analyzed for particle size. About 10 μl of the sample was measured with a pipette and diluted with 500 μl of physiological saline; the sample was thoroughly mixed and used Mastersizer particle size analyzer (model hydro 2000MU, product of Malvern), selects the Dynamic Light Scattering (DLS) module-method for particle size analysis; uses the measurement to obtain particle size distribution data, and the particle size distribution diagram of the formulations were obtained.

### Subcutaneous Tumor Model in Nude Mice

The experimental design and the protocol of animal-related experiments, which were performed in accordance with the U.K. Animals Act, 1986 (Scientific Procedures) guidelines, were reviewed and approved by the Institutional Animal Care and Usage Committee, the Fifth Medical Center of General Hospital of Chinese PLA. For the subcutaneous tumor experiments, MHCC97-H cells were cultured and prepared as a single-cell suspension for injection subcutaneously into nude mice (5 × 10^6^ cells injected in every nude mouse) (37–39). Nude mice (BALB/c mice with the absence of thymus/T cells) aged 4-5 weeks were purchased from the Si-Bei-Fu Corporation (Beijing City, China) and reared in specific pathogen-free conditions. After 2-3 weeks, in preparation for the next step (experiments of the *in vivo* sustaining ability of sorafenib formulations), the volumes of the subcutaneous tumors reached almost 1200 mm^3^. For the intrahepatic tumor model in immunodeficient rats (40), the MHCC97-H cells were cultured and injected into the nude mice to form the subcutaneous tumor tissues. When the tumors were formed, the tumor tissues were separated and prepared as tissue micro-blocks for the next experiments.

### Release of Sorafenib From Formulations *In Vitro* or *In Vivo*

The rate of sorafenib releases from different formulations was examined by *in vitro* and *in vivo* methods. For the *in vitro* testing, Sor-MS was mixed with 10 mL of physiological saline (0.9% NaCl solution), added to 0.1% Tween 80, and analyzed by vortex shock conditions (27). A 1-mL volume of solution was removed at the indicated time points. After removal, physiological saline was added to maintain a total volume of 10 mL (27). For the *in vivo* experiments, Sor-Sol (as the control) or Sor-MS was directly injected into the subcutaneous tumors formed by MHCC97-H cells (percutaneous puncture), and tumor tissues were harvested at each time point. The physiological saline samples containing sorafenib or the tumor samples containing sorafenib obtained from these experiments were mixed with acetonitrile, and sorafenib was extracted from the samples. The amount of sorafenib release into physiological saline or the amount of sorafenib sustained in the tumor tissues at the indicated time points was identified by LC-MS/MS according to the methods described in a previous publication (38). The half-life (t_1/2_) values of sorafenib were calculated according to the methods described by Wang et al. in 2020 (41). The expression of cellular proliferation, prosurvival/antiapoptosis factors, and epithelial-mesenchymal transition (EMT)–related factors in the subcutaneous tumor tissues was examined by qPCR (qualitative polymerase chain reaction) according to the methods by Ma et al. (42), and the primers used in the qPCR also were from Ma et al. (42). The heatmap of the qPCR results was performed according to the methods of Zhou et al. (43).

For the intrheptic tumor model, the Sor-MS was directed injected into the intrahepatic lesion formed by MHCC97-H (rats are injected directly into the tumor tissue after opening the abdomen). The *in vivo* release of sorafenib from HCC tissues injected with sorafenib formulations was measured by the concentration of sorafenib in the blood of nude mice or immunodeficiency rat in **Figures 2**, **3**. At each time point, blood of animal was harvested and analyzed by the LC-MS/MS.

### Intrahepatic Tumor Model in Immunodeficiency Rats

To produce an intrahepatic tumor model (the liver *in situ* tumor model) in immunodeficient rats, the HCC cells (MHCC97-H cells) were injected subcutaneously into nude mice to form tumor tissues. Then, the micro-blocks of tumor were separated from subcutaneous tumors formed by MHCC97-H cells and directly inoculated into the livers of the immunodeficient rats (44–46). The weights of the micro-blocks were shown as **Supplemental Table 1**. After 3-4 weeks of growth, the MHCC97-H cells formed intrahepatic lesions/nodules in the rat livers, and the sorafenib formulations were directly injected into the intrahepatic lesions. At the same time, another batch of rats received oral administration of sorafenib once every 2 days. The livers were collected, and photographs were obtained and analyzed by Image J Software (Version No. 1.51j8; National Institutes of Health, Bethesda, Maryland, USA) (47). Next, the intrahepatic lesions/nodules were confirmed by the pathological analysis of Masson staining (48).

### Side Effects of Sorafenib on Animals

The side effects of sorafenib on animals were identified by searching for injury to the fundal capillary network induced by sorafenib treatment. The fundus capillary network was examined by microcirculation imaging using the Retinal Imaging System (OPTO-RIS, Optoprobe, Canada). Immunodeficiency rats were intraperitoneally injected with ion of 1% pentobarbital sodium (0.3 mL/100 g) plus sumianxin (0.05 mL, 100% concentration). After general anesthesia, compound tropicamide eye drops (with eye surface anesthesia using oxybuprocaine hydrochloride eye drops) were used to induce mydriasis. The images of the fundus and retina of rats were obtained and quantitatively analyzed by Image J (47). Moreover, the body weight, hematological parameters, and mass of the main organs of animals (nude mice or the immunodeficiency rat) were examined according to the methods descripted by Huo et al. (32).

### Statistical Analysis

All statistical significance analyses were performed using SPSS 9.0 statistical software (IBM Corporation, Armonk, New York, USA). The half-life values of the release from sorafenib formulations *in vitro* and *in vivo* were calculated with Origin 6.0 software (OriginLab, USA). Statistical significance was analyzed by Bonferroni correction with two-way analysis of variance for the groups. Paired samples were tested by paired-sample t tests.

## Results

### Preparation of the Sorafenib Formulations

**Figure 1:**
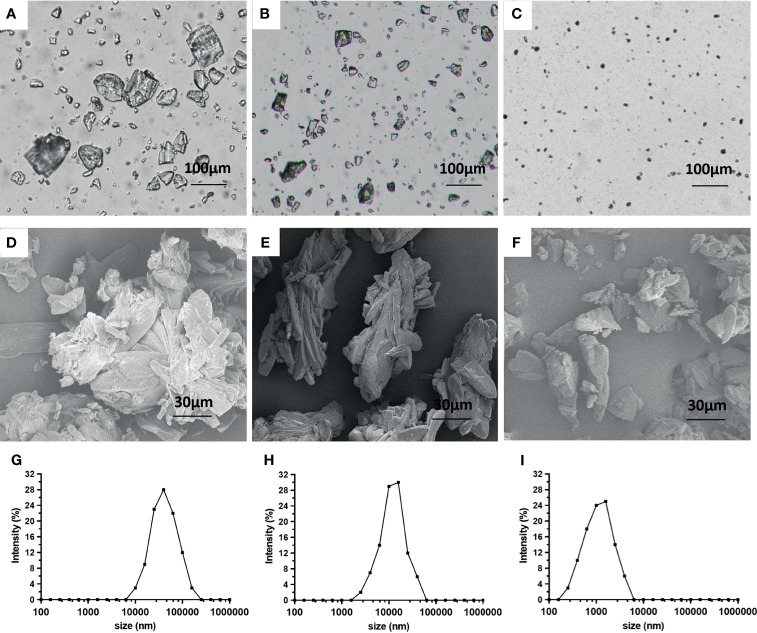
Preparation of sorafenib microcrystal formulations. The microcrystal formulations were observed by an optical microscope **(A–C)** or a conventional transmission electron microscope **(D–F)**. Particle size distribution charts of the sorafenib microcrystal formulations **(G–I)**.

First, the microcrystal and solution sorafenib formulations were prepared (**Figure 1**). The microcrystal formulations of sorafenib contained irregularly shaped crystals with varied particle diameters. According to the size of the individual particle diameter (large, medium, or small) of the sorafenib crystals, three kinds of formulations were obtained (**Figure 1**). The results were visualized as optical microscope images (**Figures 1A–C**) or transmission electron microscope images (**Figures 1B–D**) as well as particle-size distribution images (**Figures 1E–G**). Examination of the concentration of sorafenib in formulation showed that the concentration of the microcrystal formulation reached more than 30 mg/mL (**Table 1**).

**Table 1:** The concentrations of sorafenib in the formulations.

Formulations	Before filter	After filter
	concentration (mg/ml)	
Sor-Sol	2.11 ± 0.55	2.15 ± 0.28
Sor-MS small	29.79 ± 0.76	2.37 ± 0.47
Sor-MS middle	32.06 ± 0.30	2.04 ± 0.11
Sor-MS large	33.11 ± 1.08	1.93 ± 0.33

Next, the formulations of sorafenib were filtered through a 0.1-μm pore-size filter to confirm the size of the individual particle diameter of the sorafenib crystals. As shown in **Table 1**, multiple filtrations *via* 0.1-μm apertures filtrations significantly decreased the concentration of sorafenib in the microcrystal formulations but not the sorafenib solution. Moreover, there were no significant differences between the concentrations of the microcrystal formulations after filtration (**Table 1**). Multiple filtrations did not affect the concentration of Sor-Sol. Thus, the microcrystal formulations of sorafenib were successfully prepared (**Table 1**).

### Release of Sorafenib Formulations *In Vitro* or *In Vivo*

LC-MS/MS was used to examine whether the prepared sorafenib formulations could achieve long-sustaining delivery of sorafenib, and the *in vitro* or *in vivo* release of sorafenib from the formulation was revealed by t_1/2_ values (as shown in **Table 2**). The half-life values: t_1/2_ values of sorafenib microcrystal formulations by particle diameter were as follows: 360.21 ± 17.75 h (large particle diameter), 174.05 ± 7.91 h (medium particle diameter), and 51.33 ± 10.42 h (small particle diameter). The large particle diameter released sorafenib *in vitro* most slowly among the three formulations and was named Sor-MS.

**Table 2:** the *in vitro* and *in vivo* of sorafenib formulations.

Formulations	*in vitro*	*in vivo*
	half-life values (t_1/2_ [h])	
Sor-Sol	–	26.71 ± 7.44
Sor-MS small	51.33 ± 10.42	78.67 ± 9.82
Sor-MS middle	174.05 ± 7.91	240.55 ± 10.40
Sor-MS large	360.21 ± 17.75	410.36 ± 17.93

The *in vivo* releasing rates of sorafenib from the microcrystal formulations were examined by LC-MS/MS; the t_1/2_ values for the different formulations were as follows: 26.71 ± 7.44 h for the sorafenib solution formulation [Sor-Sol]), 410.36 ± 17.93 h for the sorafenib microcrystal formulation with large particle diameter, 240.55 ± 10.40 h for the sorafenib microcrystal formulation with medium particle diameter, and 78.67 ± 9.82 h for the sorafenib microcrystal formulation with small particle diameter. These results demonstrated that microcrystal formulations of sorafenib could achieve *in vitro* sustained release.

### Single Administration of Sor-MS, But Not Sor-Sol, Significantly Inhibited *In Vivo* Growth of MHCC97-H Cells

**Figure 2:**
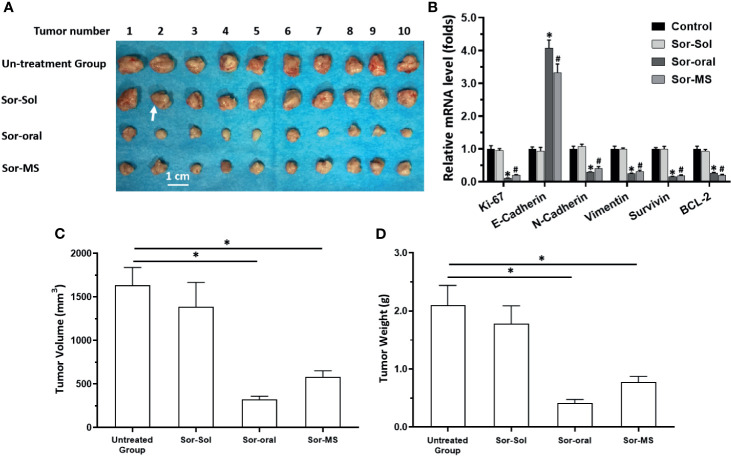
Antitumor activation of sorafenib formulations in a subcutaneous tumor model in nude mice. MHCC97-H cells were cultured and injected subcutaneously into nude mice. Mice received one intratumor injection (50μl amount) of sorafenib solution (Sor-Sol) (2mg/ml concentration), one intratumor injection (50μl amount) of sorafenib microcrystal formulation with the largest particle-size (Sor-MS) (2mg/ml), or sorafenib *via* oral administration (2mg/kg dose, repeatedly over a long period of time). Results are shown as images of subcutaneous tumor tissues **(A)**, the expression level of proliferation-related factors in the tissues **(B)**, tumor volumes **(C)**, and tumor weights **(D)**. *P < 0.05 versus untreated group with Sor-Oral group; ^#^P < 0.05 versus untreated group with Sor-MS group. The write arrow indicated the subcutaneous tumor tissues.

**Figure 3:**
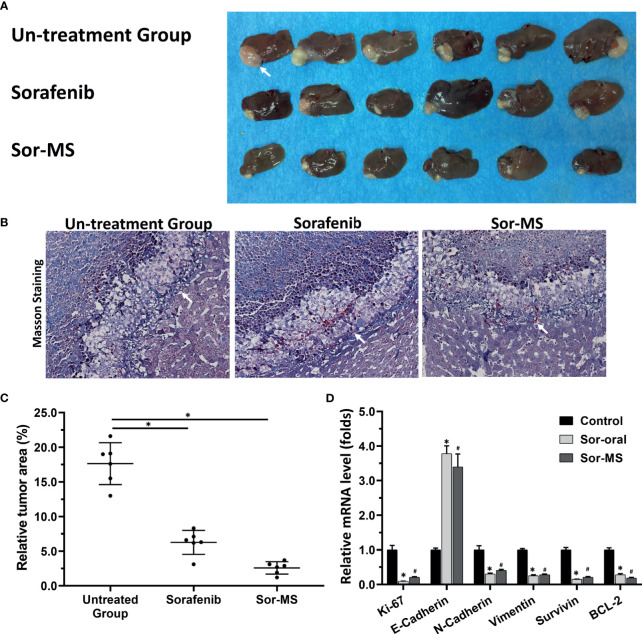
Antitumor activation of sorafenib formulations in an intrahepatic tumor model of immunodeficient rats. MHCC97-H cells were cultured, and the intrahepatic lesions of hepatocellular carcinoma (HCC) were established in the live organs of immunodeficient rats. Rats received one intratumor injection (50μl amount) of sorafenib microcrystal formulation with the largest particle-size (Sor-MS) (30mg/ml) or sorafenib *via* oral administration (2mg/kg concentration, repeatedly over a long period of time). Results are shown as images of rats’ liver organs with lesions formed by MHCC97-H cells **(A)**, the relative total area of the lesions **(C)**, and the expression level of proliferation-related factors in the tissues **(D)**. **(B)** the images from masson staining results indicated the boundary between rat liver tissue and the intrahepatic lesions. *P < 0.05 *versus* untreated group (Sor-Oral group); ^#^P < 0.05 *versus* untreated group with Sor-MS group. The write arrow indicated the intrahepatic lesions/nodules.

The “Section 3.2 results” showed that preparation of sorafenib as a microcrystal formulation could achieve sustained releasing/long-sustaining of sorafenib in tumor tissues. Whether preparation of sorafenib as a microcrystal formulation could achieve the long-acting antitumor activation was also examined in the subcutaneous tumor model. As shown in **Figure 2**, one-time intratumor injection of Sor-MS, but not Sor-Sol, could significantly inhibit the subcutaneous growth of MHCC97-H cells in nude mice. Moreover, the tumor tissues were collected and analyzed for qPCR, which showed that single administration of Sor-MS, but not Sor-Sol, could inhibit the EMT process of HCC cells in subcutaneous tumor tissues (**Figure 3**).

### Sor-MS Alleviated the Side Effects of Sorafenib in Animals

**Figure 4:**
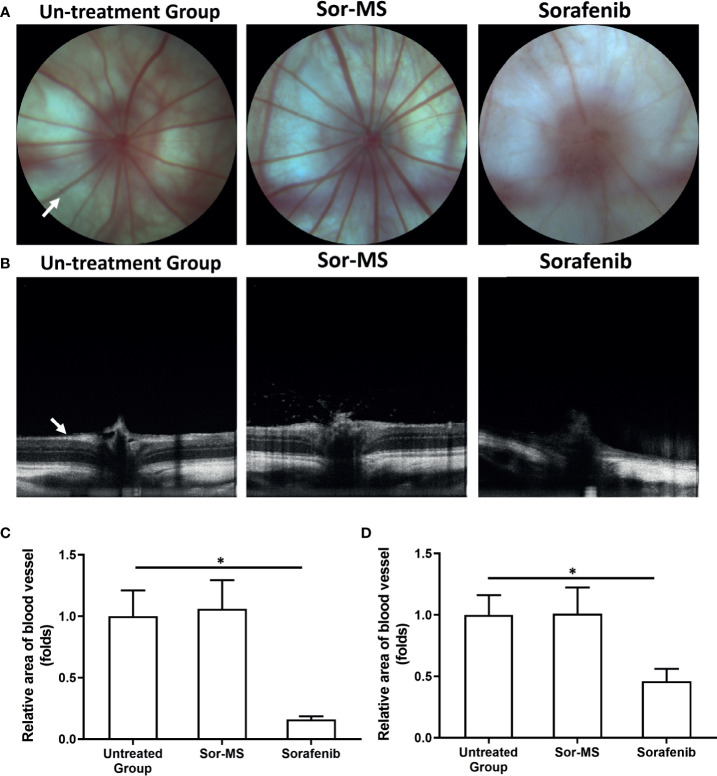
Fundus intravital imaging of immunodeficiency rats with intrahepatic lesions that received sorafenib formulations. MHCC97-H cells were cultured, and the intrahepatic lesions of hepatocellular carcinoma (HCC) were established in the live organs of immunodeficient rats. Rats received one intratumor injection (50μl amount) of sorafenib microcrystal formulation with the largest particle-size (Sor-MS) (30mg/ml) or sorafenib *via* oral administration (2mg/kg concentration, repeatedly over a long period of time). Results are shown as images of the fundus microcirculation capillary network **(A)** and fundus retinal intravital images **(B)**. Results are shown as images of the rat fundus retinal capillary network **(A)**, images of the rat fundus retinal thickness **(B)**, and a quantitative analysis of the images of the rat fundus retinal capillary network **(C)** or rat fundus retinal thickness **(D)**. *P < 0.05. The write arrow indicated the capillaries and retina.

The side effects of sorafenib were examined in the rats with the intrahepatic tumor tissues. As shown in **Figure 4**, one-time administration of Sor-MS, but not Sor-Sol, could significantly inhibit the intrahepatic growth of MHCC97-H cells in the livers of immunodeficient rats. Oral administration of sorafenib (mimicking the long cycle of clinical sorafenib treatment) could also inhibit the intrahepatic growth of MHCC97-H cells.

Next, a small animal fundus imager examined the side effects of sorafenib formulations in the microcirculation of the rats. As shown in **Figure 4**, oral administration of sorafenib could significantly disrupt the microcirculation and decease the retinal thickness of rats compared with the untreated group. Conversely, single administration of Sor-MS did not disrupt the microcirculation and decrease the retinal thickness of rats compared with the untreated group or the group that received sorafenib orally. The effect of sorafenib’s formulation of the body weight, hematological parameters, and mass of the main organs of animal were also examined to further reveal the adverse effects induced by sorafenib. As shown in **Tables 3** and **4**, oral administration of sorafenib (Sor-Sol), but not intra-tumor injection of Sol-Sol or Sor-MS, significantly induced the decrease in hematological parameters (leukocyte, Red blood cell, Hemoglobin or Platelet count), body weight, or the major organs (heat, liver, lung, kidney or spleen) of the nude mice mentioned in **Figure 2**. Moreover, it is worth noting that the oral administration of sorafenib for a long-term induced the serious injury of immunodeficiency rats’ hematological parameters, body weight, and weights of major organs mentioned in **Figures 3** and **4** (**Tables 5** and **6**). A single intra-tumor injection of Sor-MS not only exerted the antitumor activation on the intrahepatic growth of HCC cells, but also did not affect the hematological parameters, body weight, and weights of major organs of immunodeficiency rats mentioned in **Figures 3**, **4** (**Tables 5** and **6**). Therefore, the Sor-MS preparation of sorafenib could improve the side effect profile of sorafenib.

**Table 3:** The effect of sorafenib formulations on nude mice’s body weight and main organs mass.

Main organs mass	Control	Sol-Sol	Sol-Oral	Sol-MS
Body weight (g)	21.15 ± 1.92	20.20 ± 2.09	11.86 ± 2.81	21.44 ± 2.45
Heart (mg)	110.79 ± 6.63	108.44 ± 1368	59.93 ± 2.80	109.65 ± 7.18
Liver (mg)	677.05 ± 37.09	658.20 ± 21.15	364.60 ± 7.56	660.00 ± 43.11
Spleen (mg)	17.64 ± 0.43	17.11 ± 0.83	9.45 ± 0.78	16.57 ± 0.27
Double kidney (mg)	257.92 ± 8.45	248.21 ± 11.94	169.06 ± 20.49	250.27 ± 16.11
Lung (mg)	176.37 ± 46.73	181.67 ± 33.29	115.02 ± 12.56	165.89 ± 18.44

**Table 4:** The effect of sorafenib formulations on nude mice’s hematological parameters.

Hematological parameters	Control	Sol-Sol	Sol-Oral	Sol-MS
Leukocyte (10^9^/L)	3.98 ± 0.78	3.99 ± 0.93	1.35 ± 0.33	3.86 ± 0.70
Red blood cell (10^12^/L)	10.14 ± 0.64	9.81 ± 0.32	4.94 ± 0.15	9.46 ± 0.34
Hemoglobin (g/L)	157.16 ± 26.65	143.12 ± 19.83	79.44 ± 8.76	150.40 ± 16.14
Platelet count (10^9^/L)	678.86 ± 49.92	618.66 ± 32.90	319.17 ± 27.38	680.54 ± 36.18

**Table 5:** The effect of sorafenib formulations on immunodeficiency rat’s body weight and main organs mass.

Hematological parameters	Control	Sor-Oral	Sor-MS
Body weight (g)	287.91 ± 42.15	185.02 ± 5655	290.08 ± 32.94
Heart (mg)	1.17 ± 0.18	0.74 ± 0.05	1.10 ± 0.14
Liver (mg)	3.72 ± 0.20	1.81 ± 0.28	3.66 ± 0.31
Spleen (mg)	0.71 ± 0.10	0.32 ± 0.08	0.67 ± 0.22
Double kidney (mg)	0.85 ± 0.22	0.43 ± 0.09	0.87 ± 0.07
Lung (mg)	3.80 ± 0.39	1.59 ± 0.62	3.94 ± 0.55

**Table 6:** The effect of sorafenib formulations on immunodeficiency rat’s hematological parameters.

Main organs mass	Control	Sor-Oral	Sor-MS
Leukocyte (10^9^/L)	10.54 ± 1.73	4.14 ± 0.69	11.30 ± 2.46
Red blood cell (10^12^/L)	7.19 ± 2.24	2.98 ± 0.81	7.59 ± 2.71
Hemoglobin (g/L)	130.19 ± 21.1	78.26 ± 6.29	125.35 ± 8.36
Platelet count (10^9^/L)	310.72 ± 25.27	184.01 ± 42.12	305.52 ± 27.38

### The Blood-Concentration of Sorafenib Released From Sorafenib

**Figure 5:**
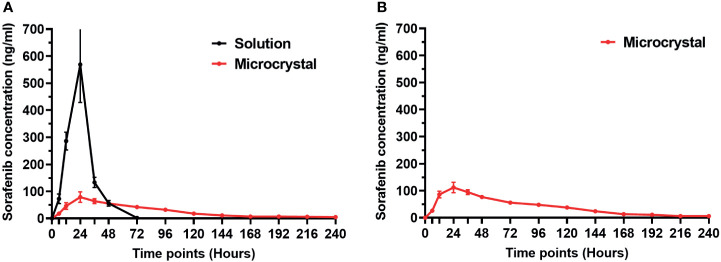
The *in vivo* release of sorafenib from HCC tissues injected with sorafenib formulations. The *in vivo* release of sorafenib from HCC tissues injected with sorafenib formulations was measured by the concentration of sorafenib in the blood of nude mice **(A)** or immunodeficiency rat **(B)** in Figures 2 and 3. At each time point, blood of animal was harvested and analyzed by the LC-MS/MS. The images are shown as the blood-concentration-curve of sorafenib. Solution refers to Sor-Sol and microcrystal refers to Sor-MS.

Although the calculating the half-life values in tumor tissue could reflect the metabolism and clearance rate of sorafenib, it is still insufficient. Therefore, the concentration of sorafenib in the blood of animals after intra-tumor injection of sorafenib formulations was further examined by LC-MS/MS. As shown in **Figure 5A**, after injection of Sol-Sol in nude mice, sorafenib was rapidly cleared from the subcutaneous tumor tissues, and its blood concentration peaked within 24h. However, after intra-tumor injection of Sor-MS, the clearance rates of sorafenib from the tumor tissues was much slower compared with Sor-Sol, and the concentration of sorafenib in nude mice’s blood was constantly low and could be detected at the 240h time point after injection. Similar results were obtained from the intra-tumor injection of Sor-MS in immunodeficiency rats’ intrahepatic lesions (**Figure 5B**). These results further confirmed the *in vivo* long-sustaining feature of Sor-MS.

## Discussion

The molecularly targeted agents represented by sorafenib remain the first-line choice to treat advanced HCC (49–51). Although some clinical trials have shown that the oral administration of sorafenib (as NATCO) could improve the survival of patients, the side effects in these trials cannot be ignored (52). As research has progressed, some new molecularly targeted drugs, including regorafenib (53), lenvatinib (54, 55), and cabozantinib (56), have been approved to treat advanced HCC. These drugs have better therapeutic effects than sorafenib in advanced HCC (53–56). Nevertheless, these drugs are similar in structure to sorafenib and have the general structural formula of 1-(4-(pyridin-4-yloxy)phenyl)urea. Thus, these drugs may not be able to completely overcome many of the shortcomings of sorafenib. Improvements in the pharmaceutical preparation process for sorafenib will help achieve better therapeutic effects and use a different strategy than pure compound structure modification (57). To overcome the challenges associated with sorafenib administration/application, we prepared a novel formulation of sorafenib based on its insoluble features that could be easily administered into a tumor and that offered sustained-release of sorafenib in HCC tissues. One-time administration of Sor-MS achieved antitumor activation of sorafenib. This work extended our knowledge about sorafenib, and injection of Sor-MS into HCC tissues of patients, guided by computed tomography or digital subtraction angiography, would be a promising strategy for advanced HCC treatment.

Interventional therapy and molecularly targeted therapy are both treatment strategies for advanced HCC (10, 58). The existing combined therapy strategy of interventional therapy and molecularly targeted therapy mainly involves patients receiving interventional therapy, such as RFA (radiofrequency ablation) or TACE (transcatheter arterial chemoembolization), and taking molecularly targeted drugs, such as sorafenib, at the same time (59–64). Although existing research shows that molecularly targeted drugs combined with interventional therapy can significantly improve outcomes in patients, the current treatment strategy still fails to fully utilize the synergistic advantages of the two treatment strategies (59–64). Interventional therapy is an ideal strategy for the comprehensive treatment of advanced HCC: (1) TACE and other drugs can enter the HCC tumor tissue directly to avoid affecting the surrounding normal liver tissue (10, 58–62); (2) RFA can directly damage the HCC tumor tissue while avoiding damage to the surrounding tissues as much as possible (59–64). These advantages make interventional therapy useful in precision drug delivery for HCC tumors, but many shortcomings to the related research remain. Only a few antitumor drugs, such as doxorubicin, are widely used (65, 66). Therefore, the results of this study of great significance: sorafenib not only has been developed into a new pharmaceutical preparation suitable for TACE but also can provide more options for safer and more effective treatment of HCC in the future.

Sorafenib and other molecularly targeted drugs have side effects, and the core mechanism of these effects is the destruction of the microcirculation (i.e., human normal vascular endothelial cells). However, there are many difficulties in related research. Experimental animals and their tissues with developed microcirculation, including the intestinal mucosa, spleen, and alveoli, can be used for side effect research. Ultrasound may be included to determine the blood supply of these organs, and H&E staining can detect the tissue microenvironment and the microstructure of the mucous membranes.

This study explored the side effects of sorafenib, and it has many advantages compared with previous research methods. In this study, a new microcrystal formulation of sorafenib was developed to simulates interventional therapy by direct injection into the tumor tissue and long-term sorafenib treatment. Sor-MS was injected directly into the tumor tissue, and a single injection had long-term antitumor activity. At the same time, the slow-release characteristics of Sor-MS ensured that sorafenib was mainly distributed in the tumor tissues and had minimal impact on normal organs. With the control (the sorafenib oral gavage treatment), sorafenib was distributed throughout the animal, and the long-term effect of this sorafenib distribution could include damage to normal organs.

As the only transparent organ of the human body, the eyeball can be directly imaged and observed. Fundus imaging can not only take pictures of the vascular network but also detect the thickness of the retina. The results of this study show that a single injection of Sor-MS into the tumor tissue will not affect the fundus microcirculation and retina of experimental animals, whereas long-term oral administration of sorafenib to animals can destroy the fundus microcirculation and retina. Therefore, this study not only expands our understanding of sorafenib-related toxicology but also provides new insights about imaging of live small animals.

Moreover, in recent years, some particle carriers with targeted drugs have been developed. For example, Shi et al. prepared an “Apatinib-loaded CalliSpheres Beads” for embolization and examined the pharmacokinetics and tumor response in a rabbit VX2 liver tumor model (67). The strategy of this study is fundamentally different from these studies: these studies must use microspheres made of polymer materials (such as CalliSpheres Beads) to physically adsorb molecular targeted drugs, and the chemical properties of molecular targeted drugs affect the drug-amount carried by microspheres; and the drug-loaded microspheres obtained in these strategies are mainly the microspheres themselves, and the drug content is limited. The microcrystalline preparation prepared in this study does not contain polymer materials, so it can achieve a dosage of more than 30mg/ml. At the same time, the particle size of the obtained Sor-MS can be controlled through the adjustment of the process, so as to realize the embolization of the blood vessel with the molecularly targeted drug itself.

## Data Availability Statement

The original contributions presented in the study are included in the article/**Supplementary Material**. Further inquiries can be directed to the corresponding authors.

## Ethics Statement

The studies involving human participants were reviewed and approved by Ethics Committee of fifth medical center of Chinese PLA. Written informed consent for participation was not required for this study in accordance with the national legislation and the institutional requirements. The animal study was reviewed and approved by Animal Ethics Committee of Fifth medical center of Chinese PLA.

## Author Contributions

HX, XY, and JW designed research. JW, RL, and YZ performed the experiments. ZM, ZS, and ZW participated in the preparation of the manuscript. HX and XY wrote the manuscript with contributions from all authors. All authors contributed to the article and approved the submitted version.

## Funding

This work was supported by grants from the National Natural Science Foundation of China (No. 81971720).

## Conflict of Interest

The authors declare that the research was conducted in the absence of any commercial or financial relationships that could be construed as a potential conflict of interest.

## Publisher’s Note

All claims expressed in this article are solely those of the authors and do not necessarily represent those of their affiliated organizations, or those of the publisher, the editors and the reviewers. Any product that may be evaluated in this article, or claim that may be made by its manufacturer, is not guaranteed or endorsed by the publisher.
