# Supplementary material for: Novel Microcrystal Formulations of Sorafenib Facilitate a Long-Acting Antitumor Effect and Relieve Treatment Side Effects as Observed With Fundus Microcirculation Imaging
Source: Front Oncol. 2021 Aug 26;11:743055. doi: 10.3389/fonc.2021.743055 (PMC8426437; doi:10.3389/fonc.2021.743055)
Supplement: Supplementary file 1 [file Table_1.doc]

Supplemental Table 1 The weight of tissue micro-lumps in the experiment of liver tumor transplantation in immunodeficiency rats

| Tumor No. | Control | Sor-Oral | Sor-MS |
| --- | --- | --- | --- |
| No. 1 | 23.29 | 24.89 | 21.68 |
| No. 2 | 27.15 | 22.38 | 25.37 |
| No. 3 | 21.53 | 24.52 | 22.84 |
| No. 4 | 23.93 | 26.98 | 25.38 |
| No. 5 | 28.15 | 24.25 | 27.54 |
| No. 6 | 23.07 | 24.38 | 25.01 |
